# Rhinitis Sicca

**Published:** 1908-01-04

**Authors:** 


### Laryngology and Rhinology.

### RHINITIS SICCA.

The function of the nose is partly that of a sentinel to warn against the inhalation of noxious vapours. Though it is generally considered by the public as the organ of smell, the olfactory area forms but a minute proportion of the entire mucous surface. Irritants are perceived by the nerve-terminals of common sensation supplied by the fifth cranial nerve, while the special olfactory nerve serves much more delicately to detect odorous substances. In this connection it is interesting to note that the current of air passes up to the olfactory area much more readily with expiration than is the case during inspiration, and it is by this path that the savours which form so important a part of the sense of "taste" reach the olfactory organ. But by far the most important function of the nose is not merely to warn, but to render the inspired air harmless and fit to come in contact with the delicate epithelium of the pulmonary alveoli. This function a healthy nose performs to perfection for an indefinite time, and without damage to itself. The mouth and pharynx can warm and moisten the air, but only for a short time and at the expense of injury to the mucous membrane. The reason for this is the plentiful supply of blood-vessels and glands to the mucous membrane of the respiratory area of the nose. These are capable of supplying such a quantity of warm fluid as to raise the air to the temperature of the body, and to fully saturate it with moisture before it reaches the pharynx; and the blood-supply is so good that the heat lost from the mucous membrane during one inspiration is replaced before the next, whereas, when air is inspired through the mouth, the parts become progressively colder and drier. The slit-like shape of the nares affords a large surface for the treatment of the inspired air; and this slit can adapt itself to circumstances, becoming narrower by engorgement of the turbinals when colder air is breathed.

The path taken by the respired air is of some interest. As the nostrils open almost directly downwards, the current of inspired air passes straight up into the middle meatus; thence some travels directly to the pharynx, but much of the air eddies downwards over the inferior turbinal, to pass finally backwards along the floor of the nose, and a sluggish stream moves upwards above the middle turbinal to reach the olfactory area. During expiration the path is much simpler, three main currents passing along each meatus; and thus, as mentioned already, air reaches the olfactory region more directly during expiration than on inspiration. When one considers the large amount of heat and moisture that have to be given up to the 500 cubic centimetres of air inhaled at each inspiration, one must marvel at the extraordinary power of self-protection which enables the mucous membrane to keep itself moist and warm. And it is no wonder that it sometimes fails in the attempt. The result is known as "rhinitis sicca," and occurs especially when the bodily energy is diminished by anæmia, dyspepsia, or general debility, or when unusually dry air is habitually breathed, as in the case of cooks or stokers. The region which suffers most is that part of the septum, about half an inch from the nostril, which forms the inner wall of the vestibule. Here the air first impinges, and here the blood-supply and the mucous glands are less abundant than in the turbinal region. The spot becomes dry, dust collects on it, and, with the mucus, forms a small, hard, firmly adherent crust; this causes irritation and consequent picking or violent blowing of the nose, and, when the crust is forcibly detached, a small erosion has occurred beneath it. Another crust forms on this, and so the erosion becomes larger and deeper, and then may involve the cartilage and eventually produce a complete perforation of the septum. Even then the trouble is not at an end, for sound healing cannot take place over the exposed edge of cartilage which, like the bone of a conical stump, projects beyond the flaps; minute crusts adhere to the margin of the hole and the erosion extends. These, the so-called "idiopathic perforations" of the septum, are by no means uncommon; the symptoms are slight, and the patient is usually quite unaware of their existence. They are often, but quite erroneously, considered to be syphilitic; the syphilitic perforation is usually more irregular in shape, and involves the bony parts of the septum, whereas these simple perforations are smooth and rounded, and concern only the triangular cartilage.

The erosions thus formed are also the commonest source of epistaxis, which in healthy persons may be due to no other cause: the hæmorrhages due to constitutional causes, such as arterio-sclerosis or the severe anæmias, usually also take place from this region. Even after severe bleeding the erosion may be very minute and difficult to detect; a vessel, or a small leash of blood-vessels, may generally be seen crossing this area, and, if the part be gently rubbed with a probe, the hæmorrhage can often be started afresh. For diagnostic purposes it is obviously useless to use the probe roughly. A moral may be drawn from these simple facts. Firstly, the bleeding spot lies so far forwards that it can be controlled by compressing the nostrils between the finger and thumb, but with more certainty if a small pledget of wool be introduced half an inch within the nostril; it is rarely necessary to plug the nose farther back than this. Secondly, these cases can be easily and effectively treated by cauterisation. The situation of the bleeding spot should be carefully noted, a pledget of wool soaked in cocaine and adrenalin applied until the hæmorrhage has ceased, and the cautery applied at a just perceptible red heat; the electric cautery is the most convenient, but a hot wire or knitting-needle does very well. For the subsequent treatment, and for the treatment of rhinitis sicca in general, the patient should be instructed to keep this part of the septum always moist with a mild ointment or bland oil; if this be regularly done, the discomfort, the erosions, and the tendency to epistaxis will cease.

